# Optimized Molecular Nucleation Behaviors in Highly Efficient Organic Solar Cells Enabled by a Bezothiophene-Based Solid Additive

**DOI:** 10.3390/polym18161939

**Published:** 2026-08-07

**Authors:** Lanxiang Yu, Hansheng Chen, Chen Xie, Qingqing Zheng, Shuyi Liu, Siyue Zhou, Xuanlin Wen, Baoshen Deng, Mengshi Fang, Shenghua Liu, Hui Liu

**Affiliations:** 1 College of New Materials and New Energies, Shenzhen Technology University, Shenzhen 518118, China; 17746676812@163.com (L.Y.); chs6042@163.com (H.C.); xiechen@sztu.edu.cn (C.X.); 202300302057@stumail.sztu.edu.cn (Q.Z.); 202300302061@stumail.sztu.edu.cn (S.L.); 202300302062@stumail.sztu.edu.cn (S.Z.); 13168751301@163.com (X.W.); 2510262047@stumail.sztu.edu.cn (B.D.); 2510262094@stumail.sztu.edu.cn (M.F.); liushengh@mail.sysu.edu.cn (S.L.); 2 School of Materials, Shenzhen Campus of Sun Yat-sen University, No. 66, Gongchang Road, Guangming District, Shenzhen 518107, China; 3 College of Urban Transportation and Logistics, Shenzhen Technology University, Shenzhen 518118, China

**Keywords:** solid additives, organic photovoltaic devices, high-efficiency, morphological regulation

## Abstract

As the most critical component of organic solar cells (OSCs), the morphology of the active layer directly dictates the photovoltaic performance of the devices. Recent studies have demonstrated that tailoring the active layer morphology using solid additives is a facile and effective strategy to boost the performance of OSCs. Herein, we design and synthesize a novel solid additive, 5-bromobenzo[b]thiophene (5-BrBT), by introducing a bromine substituent onto the common benzothiophene unit. It is found that 5-BrBT optimizes the active layer formation process by effectively prolonging the nucleation time, which facilitates more controllable molecular nucleation and subsequent crystal growth during the pre-aggregation stage, leading to a more ideal donor-acceptor phase distribution. Furthermore, the binary PM6:L8-BO organic solar cells, fabricated with the incorporation of 5-BrBT, exhibit superior charge transport properties and exciton-generation efficiency, along with significantly suppressed charge recombination behaviors. Consequently, the 5-BrBT-treated binary PM6:L8-BO-based OSCs achieve an outstanding power conversion efficiency (PCE) of up to 19.44%, accompanied by simultaneous enhancements in short-circuit current density (J_SC_) and fill factor (FF). This work provides a promising optimization strategy for achieving ideal nucleation behaviors during the bulk-heterojunction (BHJ) film processing via solid additives, which is expected to promote the development of more efficient OSCs.

## 1. Introduction

Organic solar cells (OSCs) have emerged as a promising renewable energy technology due to their lightweight, flexible, and low-cost solution-processable nature, alongside their environmental benefits [1,2,3,4]. These attributes have spurred significant interest in applications such as flexible electronics, portable wearable devices, and building-integrated photovoltaics [5,6]. To advance OSC performance, researchers worldwide are actively developing new donor-acceptor materials, optimizing device architectures, and refining active layer morphology to improve power conversion efficiency (PCE) [7,8,9,10,11]. The aggregation characteristics of donor and acceptor materials enable the tuning of molecular packing morphology and active layer film quality, which in turn exerts a decisive influence on the performance of OSCs [12,13]. Thus, achieving an ideal active layer morphology—with well-controlled domain size, a bicontinuous interpenetrating network, high phase purity, and optimal vertical phase separation—is essential for maximizing device efficiency [14,15,16,17,18,19,20].

The incorporation of high-boiling-point liquid additives, such as 1,8-diiodooctane (DIO), diiodomethane (DIM), and 1-chloronaphthalene (CN), serves as a widely employed strategy to regulate the morphology of the active layer. Nevertheless, such additives are highly dose-sensitive, which easily leads to inferior reproducibility of device performance and compromised long-term stability of devices [21,22,23,24]. In recent years, researchers have proposed the strategy of using solid additives to achieve fine control over the nanoscale morphology of the active layer in OSCs [25]. Owing to the excellent molecular compatibility and strong intermolecular interactions between solid additives and photoactive materials, ordered pre-aggregated structures can be induced, and molecular packing can be precisely modulated [26,27,28,29,30]. This promotes the efficient dissociation of excitons into free charge carriers, suppresses charge recombination losses, and thus delivers remarkable enhancement of device performance [31,32].

Based on the working principles of these methods, solid additive strategies play a crucial role in optimizing organic photovoltaic devices. For example, Zhang et al. developed a solid additive naphtho[1,2-b:5,6-b′]dithiophene (NDT), which can induce well-developed phase separation and molecular packing of active layers, enabling significantly improved photovoltaic performance [33]. In particular, the OSCs based on PM6:L8-BO treated with an NDT additive achieved a high PCE of 18.55%, which is a significant improvement in PCE of 16.41% for the control device. Chen et al. proposed a perhalogenated volatile solid additive, 2,5-dichloro-3,4-diiodothiophene (SA-T5), to optimize OSCs [34]. The introduction of SA-T5 can optimize the morphology of the active layer, thereby helping to suppress charge recombination and enhance charge transportation. Compared to the as-cast devices, SA-T5-treated devices showed a remarkable PCE of 18.8%, along with improved short-circuit current density (J_SC_) and fill factor (FF). Notably, existing solid additive strategies are yet to be fully optimized: poor regulation of disordered aggregation causes excessive domain coarsening and severe charge recombination, while over-aggregation disrupts bicontinuous transport pathways and hinders exciton diffusion and dissociation. Thus, to further elevate OSC performance, it is critical to deeply investigate and elucidate the balancing mechanism of solid additives in modulating the aggregation process.

In this work, we introduced 5-bromobenzothiophene (5-BrBT) as a novel solid additive to optimize the bulk heterojunction (BHJ) morphology and enhance the photovoltaic performance of OSCs. The PM6:L8-BO blend represents a classic and highly efficient binary system in organic photovoltaics and has been extensively utilized for the fabrication of high-performance photovoltaic devices as well as mechanistic investigations of morphology regulation [35,36,37,38]. Accordingly, this work adopts the PM6:L8-BO binary bulk heterojunction photoactive layer system, where PM6 serves as the polymer electron donor, and L8-BO acts as the non-fullerene small-molecule acceptor, blended at a fixed mass ratio of 1:1.2 (*w*/*w*). It is demonstrated that 5-BrBT effectively prolongs the pre-aggregation window during film formation, modulates the nucleation and aggregation kinetics of the PM6:L8-BO blend system, and mildly promotes the molecular aggregation of the L8-BO acceptor. This contributes to the construction of a more uniform donor-acceptor (D-A) heterojunction interface and yields a well-balanced bicontinuous phase separation morphology with optimized molecular packing. Compared with conventional liquid additives such as CN, DIO, and DIM, 5-BrBT can more effectively suppress the leakage current of devices and reduce carrier recombination losses, while significantly improving the charge transport kinetics of PM6:L8-BO-based organic solar cells (OSCs), which provides core kinetic support for the enhancement of their photovoltaic performance. Consequently, the 5-BrBT-incorporated PM6:L8-BO-based OSCs achieved a remarkable power conversion efficiency (PCE) of 19.44%, outperforming the pristine control device without additive (PCE = 18.36%), with simultaneous improvements in both fill factor (FF) and short-circuit current density (J_SC_). The strategy proposed in this work is simple and efficient, and holds great promise for the development of high-performance organic solar cells.

## 2. Materials and Methods

### 2.1. Materials

PNDIT-F3N, the polymer donor PM6 and non-fullerene acceptor L8-BO were purchased from Solarmer Materials Inc., Beijing, China. 1,8-diiodooctane (DIO), diiodomethane (DIM), 1-chloronaphthalene (CN), 5-bromobenzothiophene (5-BrBT) and chloroform (CF) were purchased from Aladdin Inc., Shanghai, China. 2PACz was obtained from Tokyo Chemical Industry Co., Ltd., Tokyo, Japan. Indium-tin oxide (ITO) glass was gained from Liaoning Youxuan New Energy Technology Co., Ltd., Liaoning, China.

### 2.2. Preparation of Devices

Organic solar cell devices were fabricated with the structure of ITO/2PACz/PM6:L8-BO/PNDIT-F3N/Ag. The patterned ITO-coated glasses were sequentially ultrasonicated with detergent, deionized water, acetone, and isopropanol for 15 min each and then dried in an oven at 80 °C for 12 h. After drying, the ITO glass was treated with 6 min of plasma at a power of 170 W. Then, the substrates were transferred to the N_2_-filled glove box. 2PACz solution (0.3 mg mL^−1^ in ethyl alcohol) was subsequently spin-coated on the ITO substrate at 3000 rpm for 30 s, and the substrate was then thermally annealed at 100 °C for 5 min. For active layer fabrication, the PM6:L8-BO blend (1:1.2, *w*/*w*) was dissolved at a total concentration of 16 mg mL^−1^ either in chloroform supplemented with 0.5 vol% CN, 0.25 vol% DIO, or 0.25 vol% DIM for the control systems, or in pre-prepared 5-BrBT chloroform solutions (4.8, 6.4, 8.0, and 9.6 mg mL^−1^) for the 5-BrBT-modified systems. All solutions were stirred at 50 °C for 6 h, spin-coated onto the aforementioned substrates at 3000 rpm for 30 s, and then subjected to thermal annealing at 100 °C for 5 min. Then, a PNDIT-F3N methanol solution (0.5 mg mL^−1^) was spin-coated on top of the active layer at 3000 rpm for 30 s. Finally, the top electrode was deposited onto the PNDIT-F3N layer under 1.0 × 10^−4^ Pa in an evaporation chamber. The thicknesses of the hole transport layer, active layer, electron transport layer, and top electrode are 30 ± 3 nm, 100 ± 5 nm, 40 ± 3 nm, and 100 nm, respectively.

### 2.3. Plasma Treatment

The plasma activation treatment was performed using a PT-30SS plasma system (Shenzhen Sanwa Boda Mechanical & Electrical Technology Co., Ltd., Shenzhen, China). Oxygen was used as the process gas at an inlet flow rate of 60 mL/min. The radio frequency (RF) power was activated once the chamber pressure was reduced to 35 Pa, followed by a 40 s equilibration period.

### 2.4. Optical Characterizations

The current density-voltage (J-V) characteristics were measured by a Keithley 2400 Source Meter under simulated solar light (100 mW/cm^2^, AM1.5 G, Sirius-SS150A, Beijing Zolix Instruments Co., Ltd., Beijing, China). The EQE spectra were recorded on an EQE measurement system (Enlitech, QE-R3011, Shengyan Electronic Technology Co., Ltd., Shanghai, China). UV-Vis absorption spectra were recorded by a Lambda365 spectrophotometer (PerkinElmer, Waltham, MA, USA).

### 2.5. AFM Characterizations

Surface characteristics of the fabricated films were measured by atomic force microscopy (AFM) using a Bruker Nanoscale V station (bruker Nano Surfaces & Metrology, Goleta, CA, USA) in tapping mode.

### 2.6. DSC Characterizations

PM6:L8-BO blend films for DSC characterization were fabricated on glass substrates by spin-coating under identical processing conditions as those used for device fabrication, and were scraped off and collected after thermal annealing. The test was conducted using a METTLER TOLEDO DSC3 Differential Scanning Calorimeter (Mettler-Toledo International Inc., Greifensee, Switzerland) over a temperature range of 0–300 °C with two thermal cycles: the sample was first cooled to 0 °C, then heated to 300 °C at a rate of 10 °C/min, and subsequently cooled back to 0 °C to complete one cycle. The second heating scans (from the second thermal cycle) were adopted for analysis to eliminate the thermal history introduced during film fabrication and reflect the intrinsic thermal properties of the materials.

### 2.7. Transient Photovoltage (TPV) and Transient Photocurrent (TPC) Measurements

TPV tests were carried out with devices biased under a background light matching the open-circuit voltage under 1 sun illumination, using 8 ns, 518 nm laser pulses (spectral width 3 nm) for excitation, with signals recorded at open circuit. TPC signals were collected at short circuit with the same excitation wavelength and no background light bias.

### 2.8. Film-Depth-Dependent Light Absorption Spectroscopy (FLAS)

FLAS analysis was performed using a low-energy in situ spectroscopic etching system (PU100). Controlled etching was conducted at an RF power of 120 W and a pressure of 8 Pa, with a rate of 0.8 nm/s, and the soft ion source causes no damage to the underlying material. A fiber-coupled spectrometer (350–850 nm, 1 nm resolution) was used for in situ real-time monitoring during the layer-by-layer etching process to collect absorption spectra. Combined with the Beer–Lambert law, depth-resolved absorption spectra of the film were extracted.

In FLAS measurements, the exciton formation process is characterized by the absorption coefficient, which can be derived based on

(1) α=4πk/λ

(2) $$I\approx I_{0}e^{-\alpha (\lambda )d}$$

(3) $$A(\lambda )=lg(I_{0}/I)=-lg(e^{-4\pi kd/\lambda })$$

where α is the absorption coefficient (cm^−1^), k is the extinction coefficient (imaginary part of the complex refractive index, dimensionless), λ is the incident wavelength (nm), I_0_ and I are the incident and transmitted light intensities (W cm^−2^), d is the active layer thickness (nm), and A(λ) is the wavelength-dependent absorbance (dimensionless). For clarity, the real part of the complex refractive index (n) is fixed at 2; k is derived from α and A and varies with film depth [39,40,41].

## 3. Results and Discussion

### 3.1. Device Structures and Photovoltaic Performance

**Figure 1:**
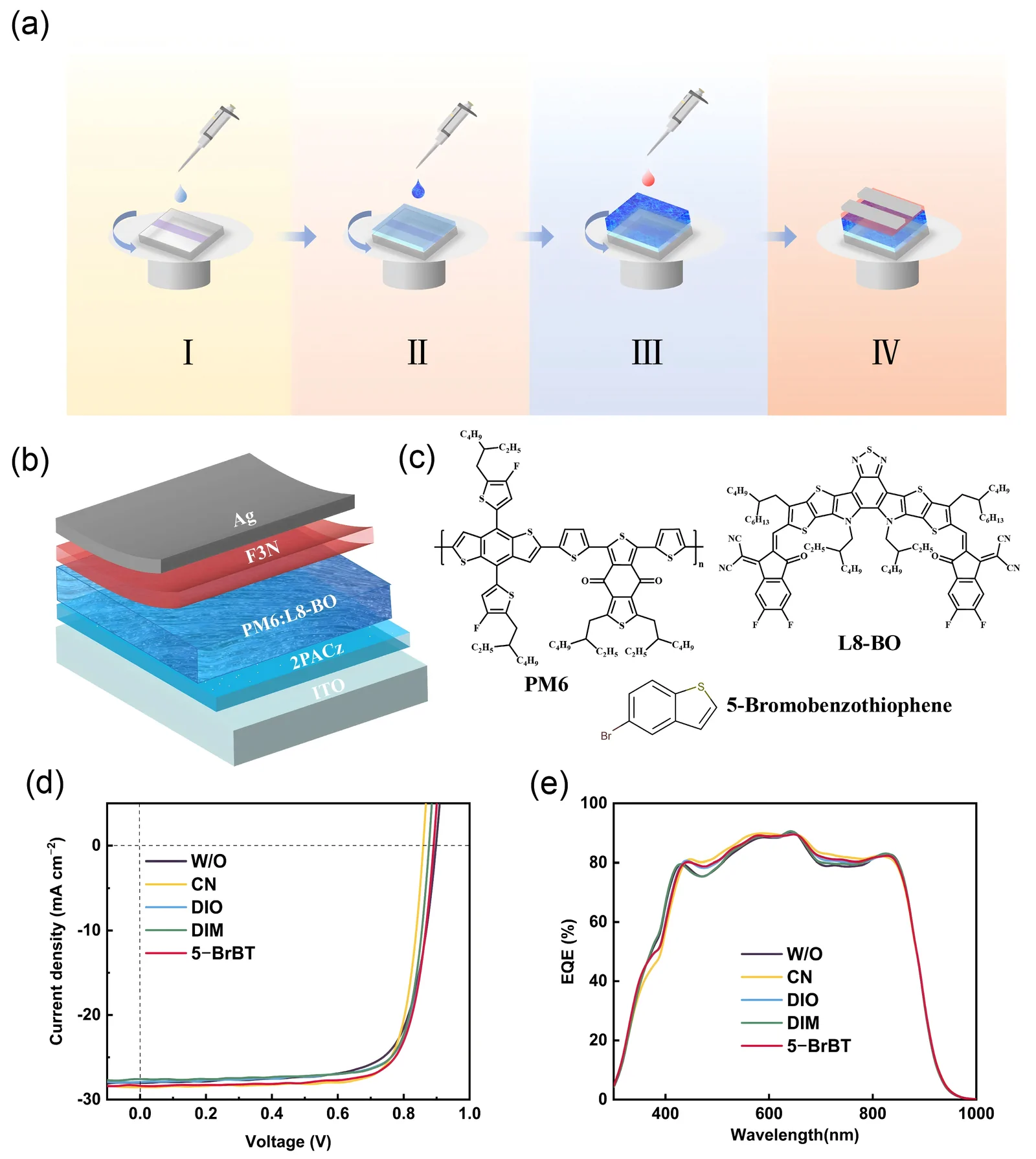
(**a**) The schematic illustration of OSC preparation. (**b**) The structure diagram of the OSC device in this work. (**c**) Molecular structures of PM6, L8-BO and 5-BrBT. (**d**) J-V curves and (**e**) EQE spectra of the OSCs based on pristine PM6:L8-BO films and PM6:L8-BO films with CN, DIO, DIM, and 5-BrBT as additives, respectively.

The fabrication process of the organic solar cells (OSCs) in this work is schematically illustrated in Figure 1a. Three functional layers are sequentially processed via spin-coating: the 2PACz hole-transporting layer in Stage I, the PM6:L8-BO active layer in Stage II, and the PNDIT-F3N electron-transporting layer in Stage III. The silver (Ag) top electrode is subsequently deposited by vacuum thermal evaporation, and the stacked architecture of the as-fabricated device is presented in Figure 1b. The binary PM6:L8-BO blend serves as the photoactive layer, where PM6 acts as a wide-bandgap fluorinated benzodithiophene-based polymer donor and L8-BO functions as a Y-series narrow-bandgap fused-ring non-fullerene acceptor [42,43,44]. 5-Bromobenzo[b]thiophene (5-BrBT) is a brominated benzothiophene derivative with a fused aromatic heterocyclic structure, as shown in Figure 1c. As confirmed by Shi et al., 5-chlorobenzothiophene with a favorable spatial electrostatic potential distribution effectively balances donor-acceptor crystallization kinetics and optimizes blend film phase separation morphology [45]. In line with the well-established elemental trend of halogen bond (σ-hole) interactions, which shows that bromine exhibits a stronger positive electrostatic potential than chlorine, 5-bromobenzothiophene can theoretically further strengthen intermolecular interactions with active layer components and achieve more pronounced crystallization regulation [46]. Accordingly, 5-BrBT is employed as the additive investigated in this work. The optimal contents of solid additive 5-BrBT in the PM6:L8-BO-based OSCs are summarized in Figure S1 and Table S1 (Supporting Information). The optimal current density-voltage (J-V) curves and external quantum efficiency (EQE) spectra of organic solar cells with different additives are presented in Figure 1d,e, and the corresponding photovoltaic parameters are summarized in Table 1. It can be seen that PM6:L8-BO-based devices without additives exhibit a PCE of 18.36%. By contrast, devices with the liquid additives CN, DIO, and DIM show enhanced PCEs of 19.28%, 19.00%, and 18.99%, respectively, primarily due to an increase in FFs. Notably, OSCs using solid additive 5-BrBT deliver a V_OC_ of 0.889 V, a pronounced J_SC_ of 28.36 mA cm^−2^, and a similarly high FF of 77.10%, resulting in a remarkable PCE of 19.44%, which is superior to that of liquid additive-based devices. Devices with different additives have no significant effect on the shapes of the external quantum efficiency (EQE) curves. As shown in Table 1, the EQEs and calculated J_SC_ of devices with different additives are in good agreement with the results from the J-V measurements.

### 3.2. Film Morphology

Atomic force microscopy (AFM) and steady-state UV-Vis absorption spectroscopy were employed to investigate the effects of additives on surface morphology and bulk molecular aggregation state of PM6:L8-BO films. We characterized the surface morphologies of films modified with different concentrations of 5-BrBT via atomic force microscopy (AFM) (Figure S2 in the Supporting Information). At concentrations of 4.8 mg/mL^−1^, 6.4 mg/mL^−1^, 8.0 mg/mL^−1^, and 9.0 mg/mL^−1^, the root-mean-square (RMS) roughness values of the films are 0.766 nm, 0.745 nm, 0.785 nm, and 0.795 nm, respectively. RMS is an external manifestation of the microphase separation and molecular packing state of the active layer, reflecting the phase separation scale of the donor and acceptor. Excessively high RMS induces a large number of bulk defects, which in turn aggravates trap-assisted recombination and shunt leakage-current losses, ultimately leading to a reduction in device FF [47,48,49]. Finally, 6.4 mg/mLmg/mL^−1^ is determined as the optimal dosage for morphology regulation, which is consistent with the J-V test results under the concentration gradient (Figure S1 and Table S1 in the Supporting Information).

**Figure 2:**
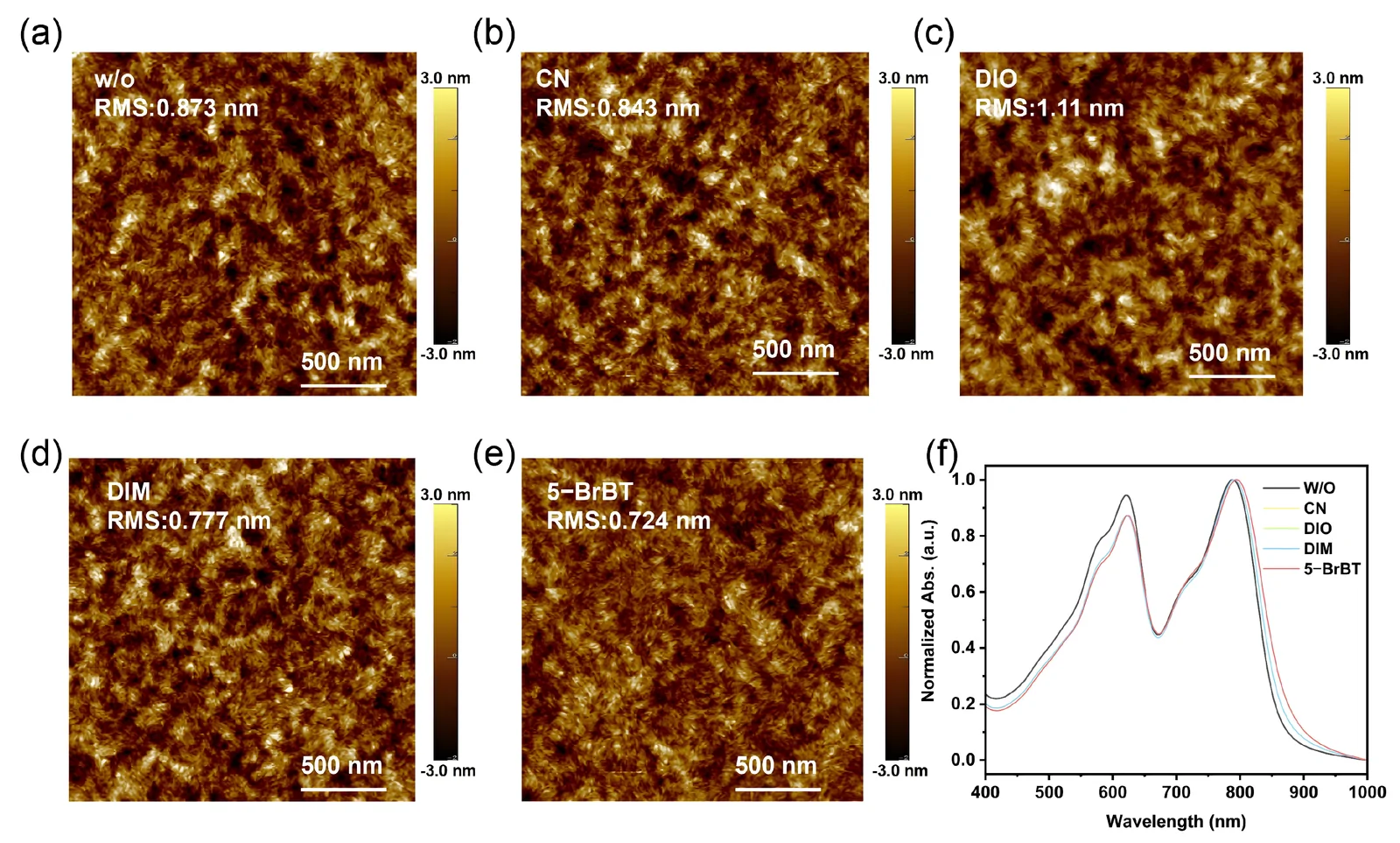
AFM height images of (**a**) pristine PM6:L8-BO film and PM6:L8-BO film with (**b**) CN, (**c**) DIO, (**d**) DIM, and (**e**) 5-BrBT as additive, respectively. (**f**) Absorption spectra of different active layers.

As shown in Figure 2a–e, the pristine film has an RMS roughness of 0.873 nm. After the introduction of CN, DIO, DIM, and 5-BrBT, respectively, the RMS roughness of the films decreases to 0.843 nm, 0.777 nm, and 0.724 nm sequentially, indicating that interfacial defects are ameliorated. The 5-BrBT-modified active layer possesses lower surface roughness and more uniform morphology. This improved surface uniformity helps reduce defect-induced trap recombination and leakage-current losses, thereby enhancing the device FF. To investigate the effect of additives on molecular structure, we systematically characterized the UV-Vis absorption properties of PM6:L8-BO films with various additives (Figure 2f). The peak positions and full widths at half maximum of the absorption spectra of PM6 films remained unchanged after modification with CN, DIO, DIM, and 5-BrBT, indicating only weak intermolecular interactions between the additives and PM6. Notably, the absorption peak of L8-BO exhibited a slight red-shift from 788 nm to 792 nm in films treated with CN, DIO, and DIM, whereas a shift to 794 nm was observed for the 5-BrBT-modified film. This result demonstrates that the solid additive 5-BrBT can mildly optimize the molecular packing of L8-BO.

**Figure 3:**
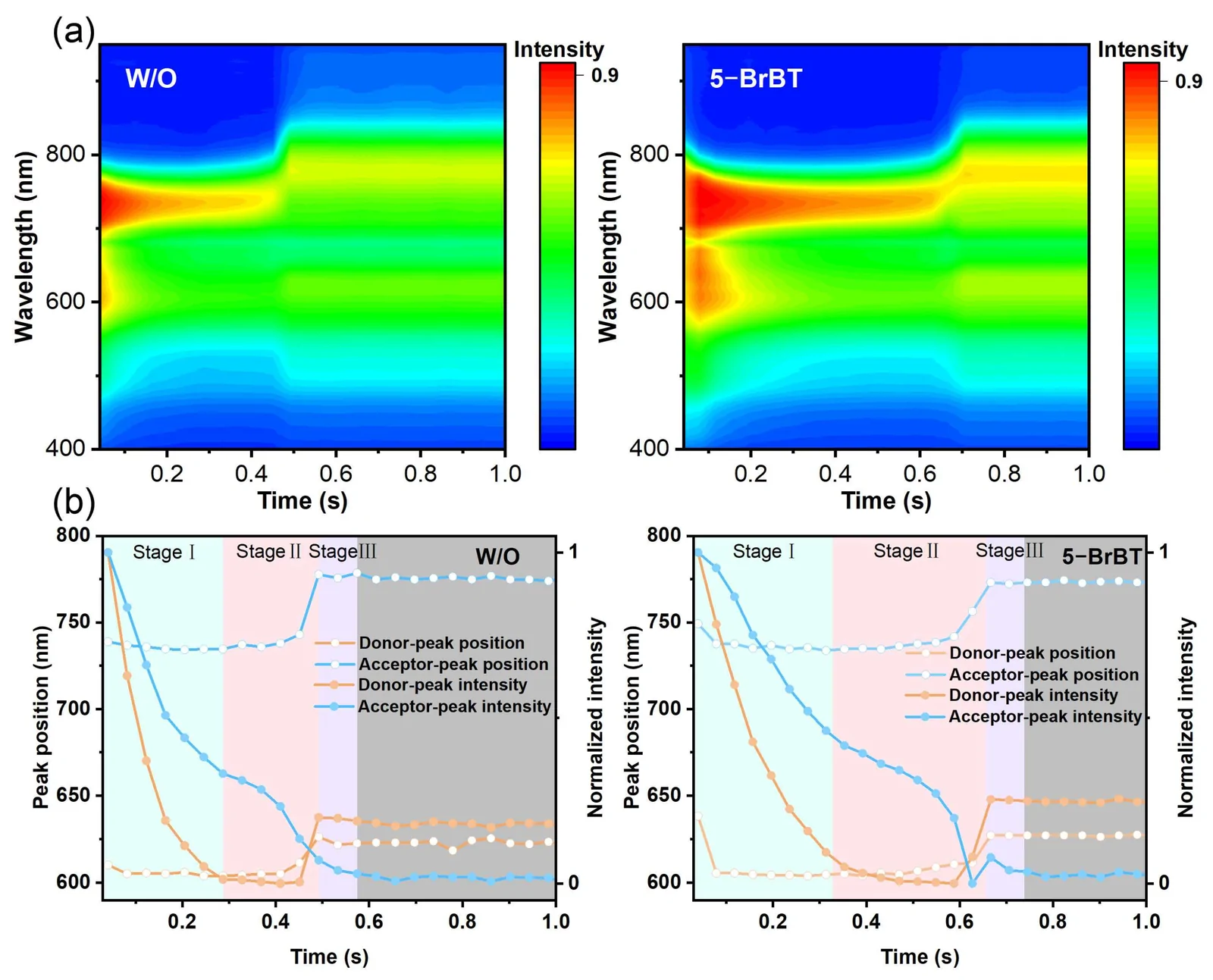
(**a**) 2D in situ UV-Vis absorption spectra, (**b**) quantitative evolution curves of absorption peak position and intensity for PM6:L8-BO blend films without additive and with 5-BrBT as the solid additive.

2D in situ UV-Vis absorption spectra were employed to investigate the crystallization kinetics of PM6:L8-BO active layers with and without 5-BrBT, and the evolution of absorption peak position and intensity during the spin-coating process is presented in Figure 3a,b and Table S2 in the Supporting Information. Based on the variation in normalized absorption intensity and the inflection points of characteristic peaks, the film formation process can be divided into three distinct stages, an analytical approach widely applied in in situ UV-Vis kinetic studies of organic photovoltaic films [50,51,52]. Stage I (solvent evaporation stage): under centrifugal force, the blend solution spreads and thins rapidly with excess solution flung off the substrate, and correspondingly, the absorption intensity drops sharply; for the pristine film without additives, this stage lasts until 0.246 s for the donor and 0.287 s for the acceptor, while after the introduction of 5-BrBT, the endpoint of this stage for the donor is extended to 0.328 s, indicating that 5-BrBT can retard the evaporation rate of chloroform and facilitate molecular diffusion. Stage II (pre-aggregation stage): the system transitions from the solution state to the film state, and the film thickness gradually stabilizes; in this stage, the decline rate of absorption intensity slows significantly, accompanied by a remarkable red-shift in the absorption peak, which reflects the progressive ordered arrangement and pre-aggregation of the blend molecules, and this stage serves as a critical window for molecular rearrangement and packing optimization; the pre-aggregation duration for the pristine film is 0.287 s for the donor and 0.246 s for the acceptor, whereas with the addition of 5-BrBT, the pre-aggregation stage for both the donor and acceptor lasts 0.451 s, and critically, this prolonged nucleation duration improves the controllability of the molecular nucleation process and exerts a positive regulatory effect on the subsequent crystal growth. Stage III (aggregation and solidification stage): the residual solvent is nearly fully evaporated, and both the absorption intensity and peak position reach a plateau without obvious changes, indicating that the molecular aggregation process is basically completed and the film structure tends to be stable; the total film formation time is 0.574 s for the pristine film and extends to 0.738 s with 5-BrBT addition.

**Figure 4:**
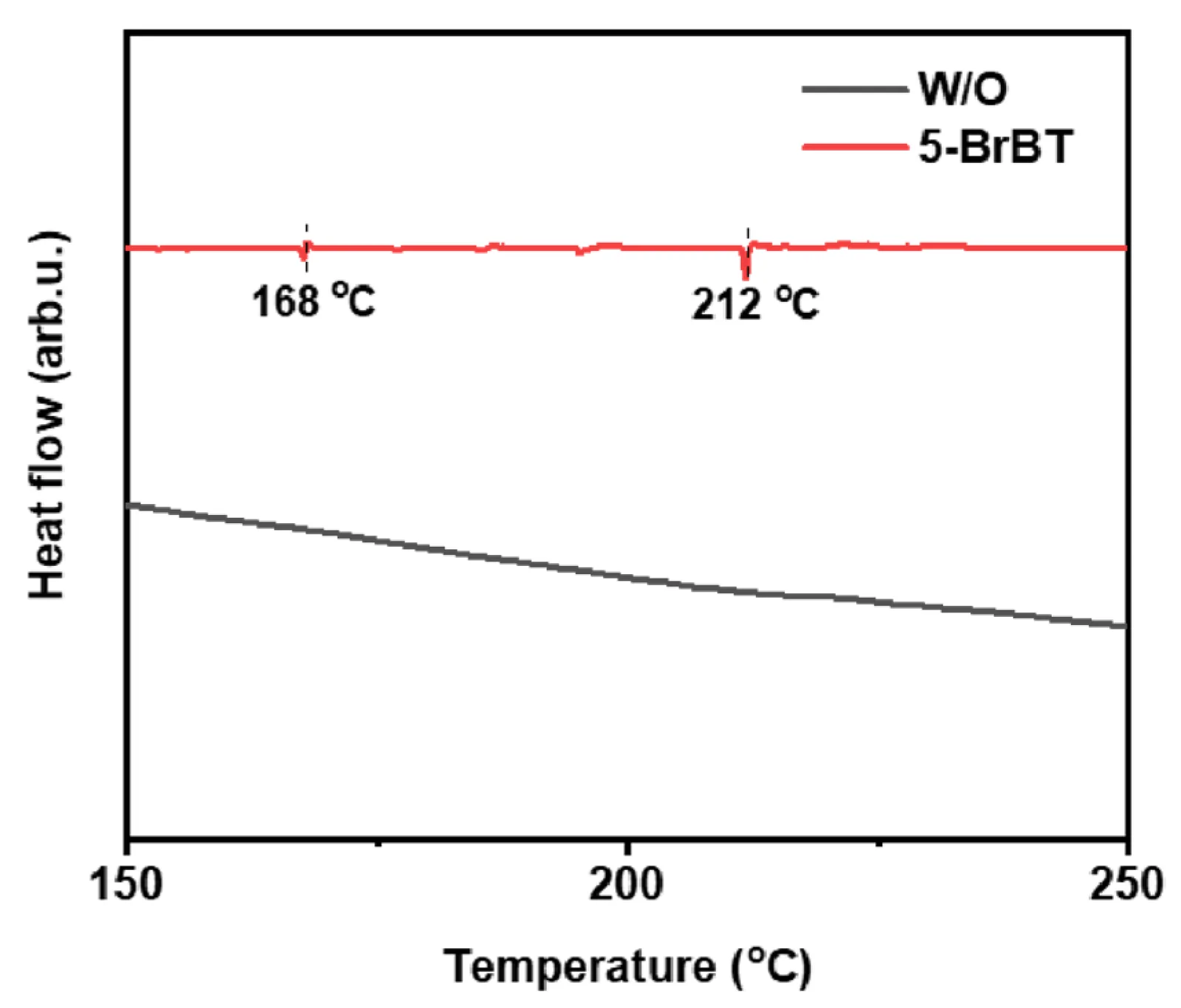
DSC curves of the PM6:L8-BO blend films without additive and with 5-BrBT as the solid additive.

The aggregation behavior of PM6:L8-BO caused by 5-BrBT was further investigated by DSC measurements. As shown in Figure 4, no evidence for any thermal transitions was observed for the PM6:L8-BO blend without additive. However, pronounced exothermic crystallization transitions of both PM6 (168 °C) and L8-BO (212 °C) were observed after addition of 5-BrBT in the active layer [53,54]. This result further confirms that 5-BrBT effectively enhances crystallization of both donor and acceptor in the PM6:L8-BO system after film formation [55,56]. In summary, the 5-BrBT additive can modulate the nucleation and aggregation kinetics of the blend system during film formation, and by retarding solvent evaporation and prolonging the pre-aggregation duration, it provides a favorable kinetic basis for constructing an ideal bicontinuous interpenetrating phase separation morphology.

**Figure 5:**
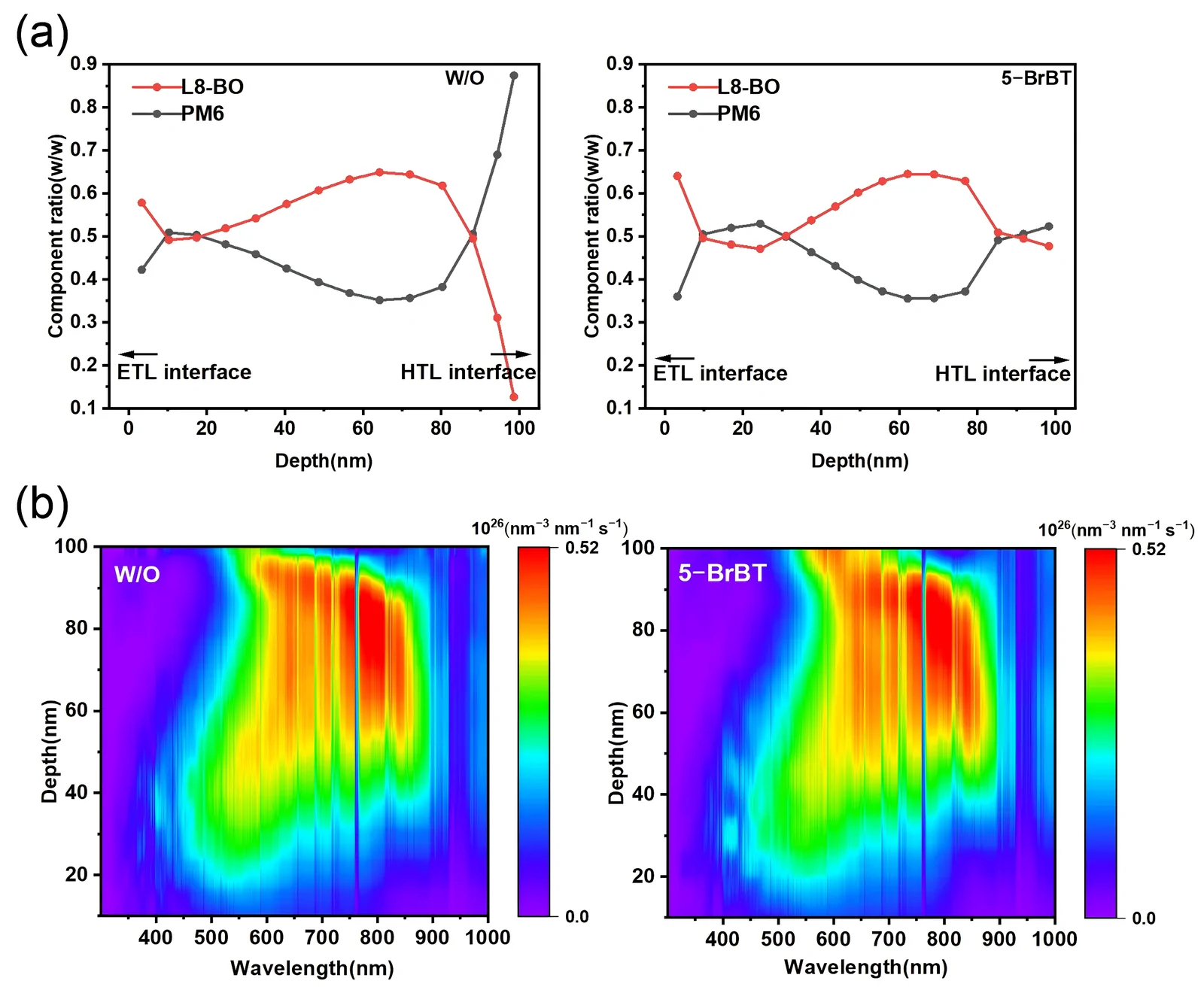
(**a**) Vertical distribution curves of component ratio, and (**b**) optical simulation contour maps of exciton-generation distribution along the thickness for PM6:L8-BO blend films prepared without additive and with 5-BrBT as the solid additive.

Subsequently, we employed film-depth-dependent light-absorption spectroscopy (FLAS), combined with soft plasma etching, to analyze the vertical phase separation characteristics of PM6:L8-BO blend films. Deconvolution was performed using depth-dependent absorption spectra and reference data of pristine PM6 and L8-BO single-component films (Supporting Information Figures S3 and S4), and the donor-acceptor composition distribution was experimentally fitted, as shown in Figure 5a. In the depth range of 80–100 nm, the pristine film exhibits a higher donor-component ratio (87.39%); however, such a composition distribution favors hole transport at the expense of exciton-generation efficiency. In contrast, the 5-BrBT-modified film achieves a relatively balanced donor-acceptor (D/A) ratio (donor ratio: 52.3%), which facilitates an optimal trade-off between exciton-generation and charge collection near the hole-transporting layer (HTL) [57,58,59,60]. Based on the depth-resolved extinction coefficients and vertical composition distribution data, simulations were carried out using the optical matrix-transfer model, and we plotted the exciton-generation contour maps along the film thickness and the variation curves of exciton-generation rate [61]. As shown in Figure 5b and Figure S5 (Supporting Information), the exciton generation rate is significantly enhanced in the region adjacent to 2PACz (0–20 nm). The precise regulation of donor-acceptor vertical phase separation in the PM6:L8-BO active layer via the 5-BrBT solid additive strategy can synergistically improve both the exciton dissociation efficiency and charge collection efficiency.

### 3.3. Device Physics Analysis

**Figure 6:**
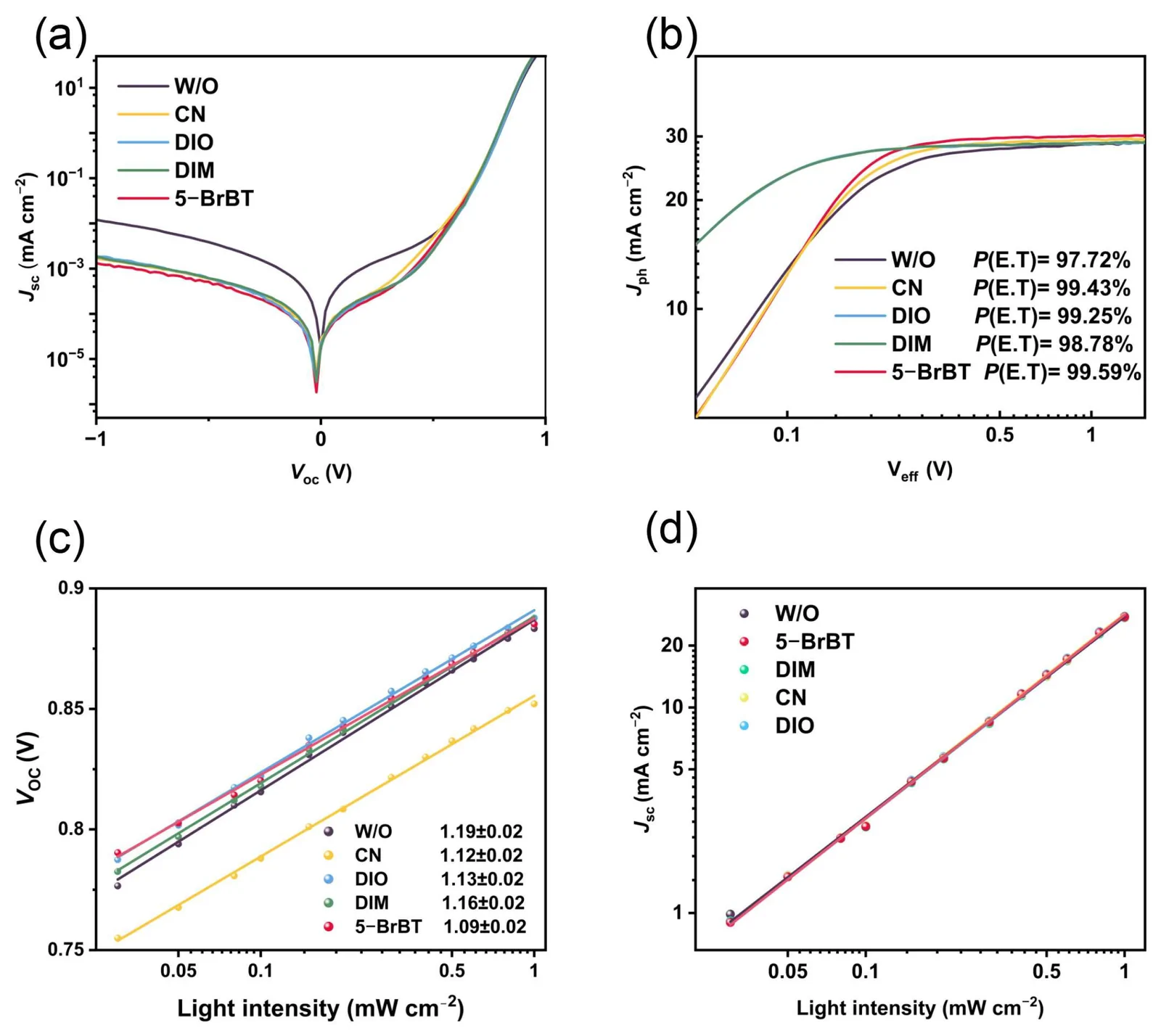
(**a**) Dark-current curves, (**b**) J_ph_ − V_eff_ curves, and light intensity-dependent characteristics of (**c**) V_OC_ and (**d**) J_SC_ of the OSCs based on pristine PM6:L8-BO films and films with CN, DIO, DIM, and 5-BrBT as additives.

Based on the as-fabricated devices, including the pristine device and devices modified with CN, DIO, DIM, and 5-BrBT additives, we first investigated their dark current density-voltage (J-V) characteristics. Dark current is the intrinsic leakage current of organic photovoltaic devices under dark conditions, which originates from non-radiative recombination and shunt leakage losses caused by defects, pinholes, and interfacial imperfections in the active layer. It is noteworthy that these defects will reduce the V_OC_ and FF of the devices. This current can preliminarily reflect the extent of carrier recombination and leakage losses within the devices [62,63]. As shown in Figure 6a, the W/O device exhibits the highest reverse leakage current (approximately 10^−2^ mA cm^−2^) at a bias of −1 V. The reverse leakage current is reduced to varying degrees in all additive-modulated devices, indicating that additives can suppress shunt loss by optimizing the active layer morphology. Among them, the 5-BrBT-modified device achieves the optimal leakage-current suppression effect, with the reverse current on the order of 10^−3^ mA cm^−2^, which can be attributed to the well-regulated active layer. In addition, we characterized the photocurrent density versus effective voltage (J_ph_ − V_eff_) curves to evaluate the exciton dissociation and charge-extraction properties of the devices. The exciton dissociation and charge collection probability P_(E,T)_ was derived from the ratio of the photocurrent density to the saturated photocurrent density (J_ph_/J_sat_), and the corresponding results are displayed in Figure 6b. The photocurrent density J_ph_ is defined as J_ph_ = J_light_ − J_dark_, where J_light_ and J_dark_ represent the short-current density under illumination and in the dark, respectively. The effective voltage V_eff_ follows the expression V_eff_ = V_0_ − V_a_, in which V_0_ is the compensation voltage at which J_ph_ equals zero, V_a_ is the applied bias, and J_sat_ refers to the saturated photocurrent density (J_ph_ value under a V_eff_ beyond 2 V) [64]. The calculated P_(E,T)_ values for the pristine device and the CN-, DIO-, DIM-, and 5-BrBT-modified devices are 97.72%, 99.43%, 99.25%, 98.78%, and 99.58%, respectively, confirming that additive modification can effectively enhance the exciton dissociation and charge collection efficiency in the active layer.

Furthermore, charge recombination dynamics were evaluated by analyzing the dependence characteristics of both V_OC_ and J_SC_ on light intensity (P_light_). The relationship between V_OC_ and Plight can be expressed as V_OC_ ∝ (nkT/q)lnP_light_, where n is the ideality factor, k is the Boltzmann constant, T is the Kelvin temperature, and q is the elementary charge, respectively. When n is close to 1, the charge recombination in a device is commonly a bimolecular recombination mechanism, whereas the charge recombination may be dominated by trap-induced recombination when n is close to 2 [65,66]. We measured the J-V characteristics of the devices across a light-intensity range from 0.01 to 1 sun and extracted the V_OC_ and J_SC_ values under different light intensities; both parameters increase monotonically with rising light intensity. As shown in Figure 6c, the linear fitting slopes (in units of kT/q) of the pristine device and the devices modified with CN, DIO, DIM, and 5-BrBT are 1.19 ± 0.02, 1.12 ± 0.02, 1.13 ± 0.02, 1.16 ± 0.02, and 1.09 ± 0.02, respectively. Compared with the pristine device, all additive-modified devices show decreased fitting slopes, indicating that additive modification can mitigate trap-assisted recombination to a certain degree [67,68]. In addition, the relationship between J_SC_ and P_light_ follows a power-law equation of J_SC_ ∝ P^α^_light_, where α is the exponential factor that can be estimated from the slope of lg(J_SC_) − lg(P_light_) fitting curves. As shown in Figure 6d, the J_SC_ of all devices increases monotonically with light intensity, and the fitting curves are nearly superimposed over the entire measurement range, which is consistent with the comparable external quantum efficiency (EQE) spectral responses across different devices. This result indicates that the additive engineering strategy does not significantly alter the intrinsic photogenerated carrier-generation capability of the active layer.

**Figure 7:**
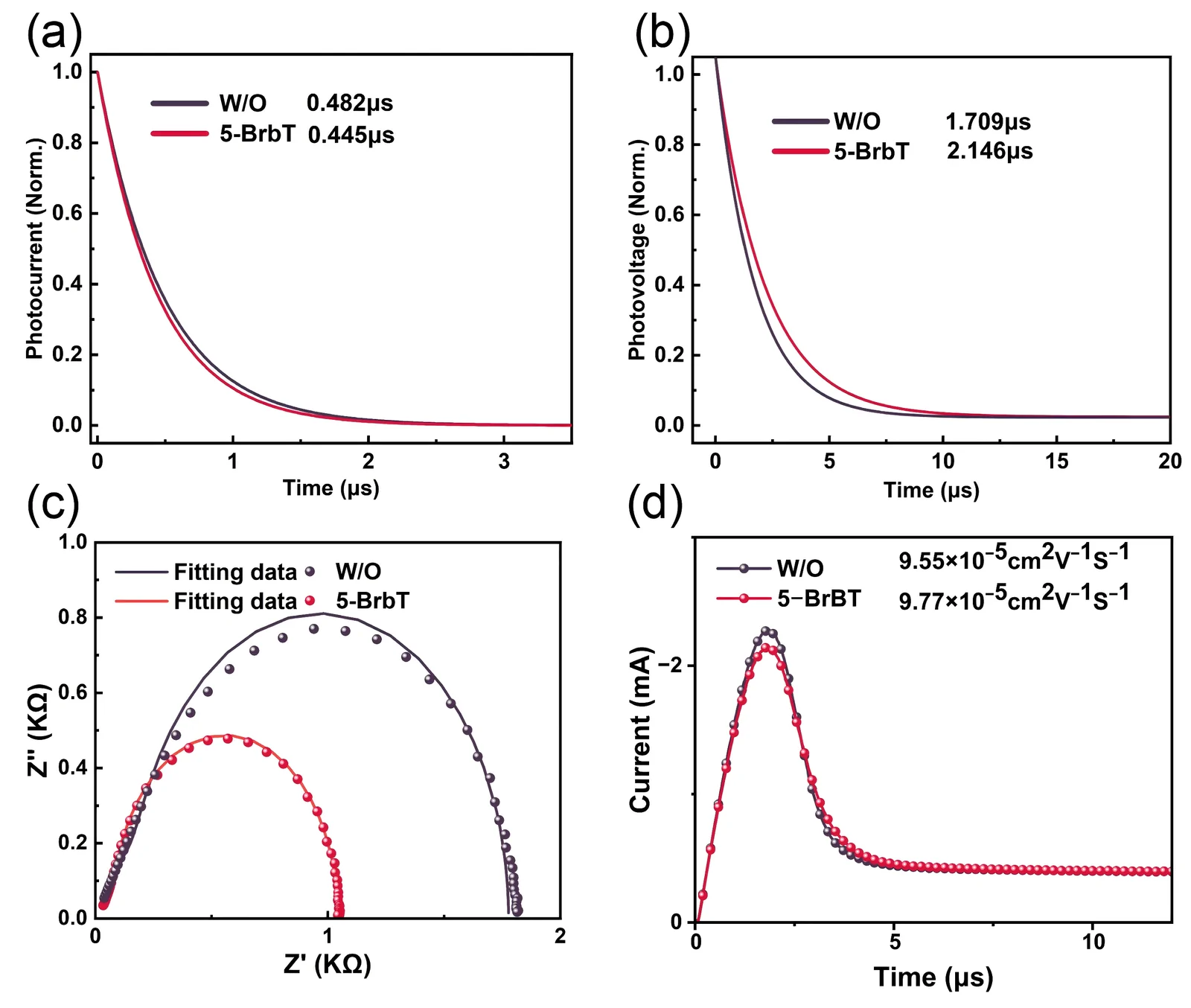
(**a**) TPC decay curves, (**b**) TPV relaxation dynamics, (**c**) Nyquist plots, and (**d**) Photo-CELIV results of the devices based on the pristine film and the film with 5-BrBT as additive.

The charge-carrier dynamics of PM6:L8-BO-based solar cells were investigated via transient photocurrent (TPC) and transient photovoltage (TPV) measurements. As shown in Figure 7a, the fitted charge-extraction lifetimes from TPC measurements are 0.48 and 0.45 μs for the pristine control (W/O) and the 5-BrBT-based device, respectively. The accelerated charge extraction by 5-BrBT constructs continuous and low-defect charge transport pathways and reduces the charge transport barrier. Meanwhile, TPV characterization was carried out to evaluate the charge recombination behavior inside the devices under open-circuit conditions. As shown in Figure 7b, the carrier lifetime significantly increases from 1.71 μs to 2.15 μs after incorporation of the 5-BrBT. These results demonstrate that the introduction of 5-BrBT is able to suppress both trap-induced and bimolecular recombination in the PM6:L8-BO-based active layer.

To investigate the charge transport dynamics of organic solar cells modified by 5-BrBT, electrochemical impedance spectroscopy (EIS) was performed under open-circuit conditions over a frequency range of 100 Hz to 1 MHz, as shown in Figure 7c. The key electrical parameters of the devices were obtained by equivalent circuit fitting (detailed in Table S3, Supporting Information). The fitting results show that, compared with the control device, the series resistance (Rs) of the 5-BrBT-modified device decreases from 31.9 to 24.1 Ω, indicating that the ohmic contact between different functional layers of the device is effectively improved. More importantly, the active medium resistance (R1) decreases drastically from 137.4 to 48.1 Ω, which confirms that 5-BrBT optimizes the bulk bicontinuous charge transport network of the active layer and significantly reduces the bulk charge transport resistance. These results provide essential electrochemical kinetic support for the enhancement of the photovoltaic performance of the devices.

Photo-induced charge extraction by linearly increasing the voltage (Photo-CELIV) measurements were conducted to investigate the charge transport behaviors. As shown in Figure 7d, the calculated average charge-carrier mobility increases from 9.55 × 10^−5^ cm^2^V^−1^s^−1^ for the control device to 9.77 × 10 × 10^−5^ cm^2^V^−1^s^−1^ for the 5-BrBT-modified device, indicating slightly improved charge transport benefiting from the optimized morphology. This result verifies that the introduction of 5-BrBT reduces the charge transport barrier and improves the carrier transport efficiency.

## 4. Conclusions

In summary, we introduced a novel solid additive, 5-bromobenzo[b]thiophene (5-BrBT), and systematically investigated its effects on active layer morphology optimization and optical property improvement when applied in organic solar cells (OSCs). During the active layer film formation process, this additive effectively prolongs the nucleation duration of the PM6:L8-BO blend system and modulates its nucleation and aggregation processes, providing a sufficient kinetic window for molecular rearrangement and packing optimization. It ultimately yields a film morphology with favorable vertical component distribution and reduced surface roughness. Through a series of device physics characterizations including light-intensity-dependent measurements, transient photocurrent (TPC), transient photovoltage (TPV), and electrochemical impedance spectroscopy (EIS), this study reveals that 5-BrBT significantly boosts exciton dissociation and charge collection efficiencies, and optimizes the carrier transport process. These improvements enable more efficient charge generation, transport, and collection in the OSCs, which ultimately contributes to a higher power conversion efficiency (PCE). With the incorporation of 5-BrBT, the champion device achieves an outstanding PCE of 19.44%, accompanied by simultaneous enhancements in both short-circuit current density (J_SC_) and fill factor (FF). The above results demonstrate that the introduction of 5-BrBT, which regulates the active layer morphology by prolonging the nucleation duration to improve device efficiency, is a feasible and effective strategy for fabricating high-performance organic solar cells.

## Figures and Tables

**Table 1:** Photovoltaic parameters of OSC devices incorporated with different additives.

Additive	*V*_OC_ [V]	*J*_SC_ [mA cm^−2^]	*J*_EQE_ [mA cm^−2^]	FF [%]	PCE [%]
W/O	0.893	28.02	26.84	73.43	18.36
CN	0.858	28.52	27.26	78.82	19.28
DIO	0.900	27.91	27.05	76.52	19.00
DIM	0.875	27.57	26.97	78.68	18.99
5-BrBT	0.889	28.36	27.12	77.10	19.44

## Data Availability

The original contributions presented in this study are included in the article/Supplementary Material. Further inquiries can be directed to the corresponding author.

## Supplementary Materials

The following supporting information can be downloaded at: https://www.mdpi.com/article/10.3390/polym18161939/s1, Figure S1: J-V curves of PM6/L8-BO-based BHJ devices fabricated with incorporation of different 5-BrBT contents; Figure S2: AFM analysis for height images and of (a) 4.8 mg mL^−1^, (b) 8.0 mg mL^−1^, (c) 6.4 mg mL^−1^, and (d) mg mL^−1^; Figure S3: (a) Absorption spectrum of neat PM6 film and (b) absorption spectrum of neat L8-BO film; Figure S4: Depth-dependent absorption spectra of ITO/PEDOT:PSS/active layer/PNDIT-F3N samples under etching time: (a) without additive, (b) 5-BrBT PM6:L8-BO films; Figure S5: The integration of exciton generation rates over film depth direction; Table S1: Photovoltaic performance of PM6:L8-BO-based BHJ devices fabricated with different 5-BrBT contents; Table S2: In situ film formation kinetic parameters extracted from time-resolved absorption spectra for PM6: L8-BO blend films with and without 5-BrBT additive; Table S3: Fitting Parameters of impedance spectroscopy using equivalent circuit.
