# Supplementary material for: Optimized Molecular Nucleation Behaviors in Highly Efficient Organic Solar Cells Enabled by a Bezothiophene-Based Solid Additive
Source: Polymers (Basel). 2026 Aug 7;18(16):1939. doi: 10.3390/polym18161939 (PMC13516338; doi:10.3390/polym18161939)
Supplement: Supplementary file 1 [file polymers-18-01939-s001.zip › polymers-4453386-supplementary.pdf]

Supporting information

Optimized molecular nucleation behaviors in high efficienct organic solar cells enabled by a bezothiophene-based solid additive

Supplementary Figure

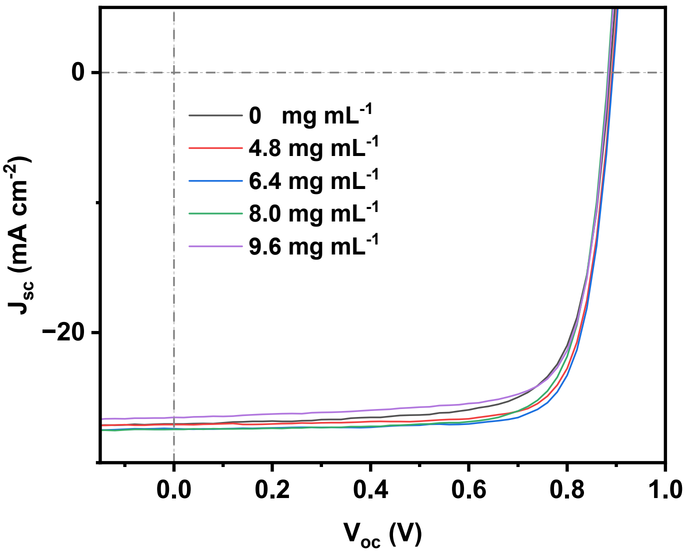

Figure S1. J–V curves of PM6/L8-BO-based BHJ devices fabricated with incorporation of different 5-BrBT contents.

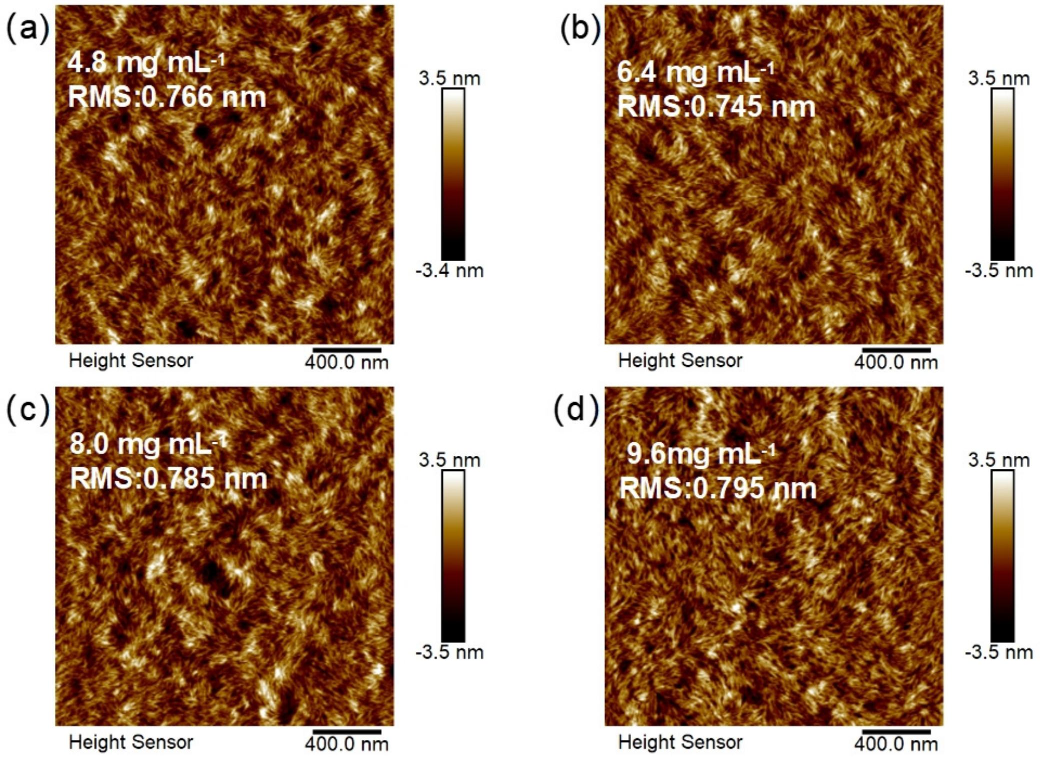

Figure S2. AFM analysis for height images and of(a) 4.8 mg mL^{-1}, (b) 8.0 mg mL^{-1}, (c) 6.4 mg mL^{-1}, and (d) 9.6 mg mL^{-1}, respectively.

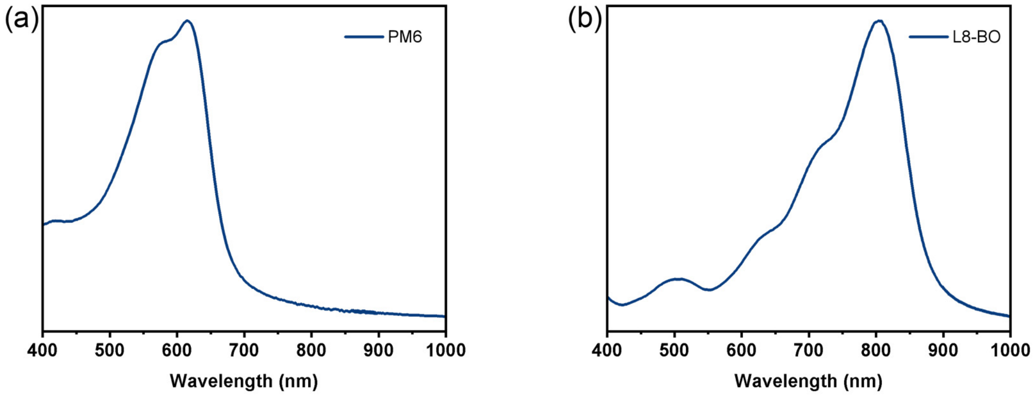

Figure S3. (a) Absorption spectrum of neat PM6 film and (b) absorption spectrum of neat L8-BO film.

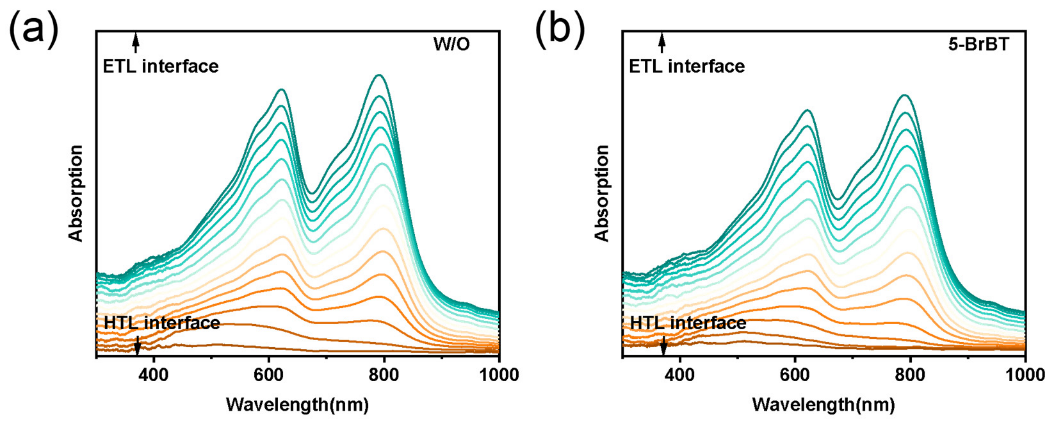

Figure S4. Depth-dependent absorption spectra of ITO/PEDOT:PSS/active layer/ PNDIT-F3N samples under etching time: (a) without additive, (b) 5-BrBT PM6: L8-BO films.

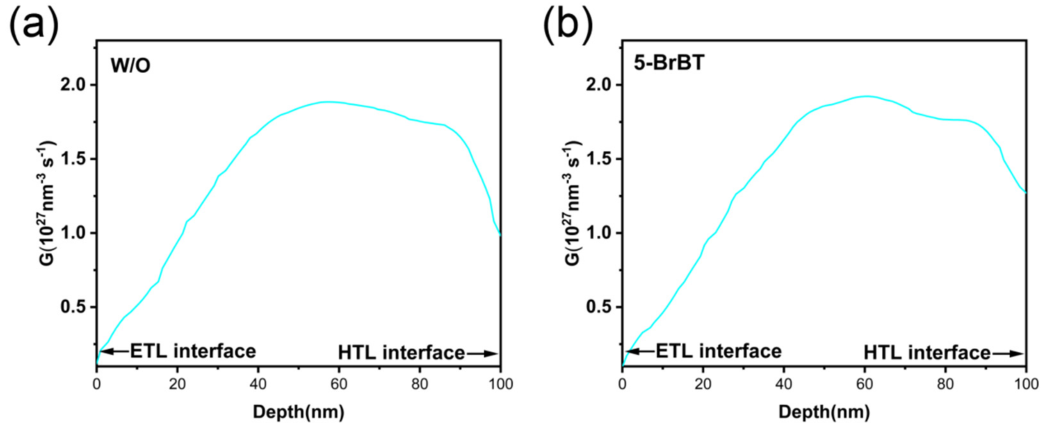

Figure S5. The integration of exciton generation rates over film depth direction.

Supplementary Table

Table S1. Photovoltaic performance of PM6:L8-BO-based BHJ devices fabricated with different 5-BrBT contents.

5-BrBT contents [mg mL^{-1}]	V_{OC} [V]	J_{SC} [mA cm^{-2}]	FF [%]	PCE [%]
0	0.885	27.01	74.30	17.77
4.8	0.890	27.07	78.24	18.84
6.4	0.891	27.39	78.70	19.21
8.0	0.881	27.44	76.84	18.59
9.6	0.882	26.54	76.07	17.82

Table S2. In situ film formation kinetic parameters extracted from time-resolved absorption spectra for PM6:L8-BO blend films with and without 5-BrBT additive.

Peak object	Additive	StageI Solvent evaporation time (s)	StageII Pre-aggregation time (s)	StageIII Aggregation time (s)	All time
Donor	Control	0.205	0.287	0.082	0.574
	5-BrBT	0.287	0.451	0.082	0.738
Acceptor	Control	0.246	0.246	0.082	0.574
	5-BrBT	0.246	0.451	0.082	0.738

Table S3. Fitting Parameters of impedance spectroscopy using equivalent circuit.

Condition	Rs [Ω]		C1 [nF]		R1 [Ω]		C2 [nF]		R2 [Ω]	
	Value [Ω]	Error [%]	Value [nF]	Error [%]	Value [Ω]	Error [%]	Value [nF]	Error [%]	Value [Ω]	Error [%]
Control	31.85	3.77	8.92	1.42	137.4	3.13	5.13	2.29	1609	0.68
5-BrBT	24.1	2.75	1.16	0.68	971	0.37	7.71	3.23	48.12	2.75
